# Development of training for medicines-oriented policymakers to apply evidence

**DOI:** 10.1186/s12961-016-0130-3

**Published:** 2016-07-29

**Authors:** H. L. Colquhoun, E. Helis, D. Lowe, D. Belanger, S. Hill, A. Mayhew, M. Taylor, J. M. Grimshaw

**Affiliations:** 1 Department of Occupational Science and Occupational Therapy, University of Toronto, 160-500 University Ave, Toronto, ON M5G 1V7 Canada; 2 Canadian Agency for Drugs and Technologies in Health (CADTH), 865 Carling Avenue, Ottawa, ON K1S 5S8 Canada; 3 Centre for Health Communication and Participation, School of Psychology and Public Health, College of Science, Health and Engineering, La Trobe University, Melbourne, Australia; 4 C.T. Lamont Primary Health Care Research Centre, Bruyère Research Institute, 43 Bruyère Street, Annex E – 208, Ottawa, ON K1N 5C8 Canada; 5 Public Health, School of Allied Health, Australian Catholic University, Fitzroy, VIC 3065 Australia; 6 Department of Medicine, University of Ottawa, School of Epidemiology, Public Health and Preventive Medicine, 451 Smyth Road, Ottawa, ON K1H 8M5 Canada

**Keywords:** Knowledge user engagement, Evidence-informed, Systematic reviews, Integrated knowledge translation, Prescribing medicines, Rx for Change

## Abstract

**Background:**

Health systems globally promote appropriate prescribing by healthcare providers and safe and effective medicine use by consumers. *Rx for Change*, a publicly available database, provides access to systematic reviews regarding best practices for prescribing and using medicines. Despite the value of the database for improving prescribing and medicine use, its use remains suboptimal. This study aimed to develop a training program for five medicine-focused organisations in Canada and Australia to facilitate the use and understanding of the *Rx for Change* database.

**Methods:**

Four steps were undertaken: 1) key informant interviews were completed across all organisations to understand the knowledge user perspective; 2) a directed content analysis was completed of the interview transcripts and proposed training was developed; 3) a second round of feedback on the proposed training by knowledge users was gathered; and 4) feedback was integrated to develop the final training.

**Results:**

Sixteen key informant interviews with knowledge users were conducted. Themes for training content included the scope of, navigation and strategies for using *Rx for Change* (generic content) and practical examples on incorporating evidence within their workplace context (tailored content). The final training consisted of an informational video, a 60-minute face-to-face workshop and two post-training reminders.

**Conclusions:**

A method of engaging knowledge users in the development of a training program to improve the use of an on-line database of systematic reviews was established and used to design training. Next steps include the delivery and evaluation of the training.

## Background

Prescribing of medications is a key element of healthcare treatment and a significant driver of healthcare costs [1]. Significant inadequacies in prescribing practices, medication errors and patient reports of prescribing errors by healthcare providers have been documented across most countries [2, 3]. In Canada and Australia, rates for medication errors are reported to be as high as 30% [2]. Medication administration and prescribing errors have been directly linked to increased morbidity [4] and other negative health outcomes such as increased infections involving antibiotic resistant micro-organisms due to overprescribing of antibiotics [5], preventable hospital admissions [6] and high rates of preventable adverse drug events [7]. Suboptimal medication use by patients is also evident, with adherence to medicines typically around 50% [8]. As such, promoting appropriate prescribing practices by healthcare professionals (providers) and appropriate medication use by patients (consumers) for maximizing patient outcomes and better managing of healthcare costs is of high interest for health systems globally [9].

Currently, a substantial evidence base about the effectiveness of various interventions for improving prescribing and medication use is available [10, 11]. Despite its availability, this evidence base is, in effect, ‘hidden’ from knowledge users (i.e. healthcare policymakers and other stakeholders interested in optimal medication use) due to its dispersion across a wide range of journals and other sources, and potential inaccessibility through the need for journal subscription. Systematic reviews, one of the best sources of research evidence, are often reported by decision-makers as not only difficult to access, but also difficult to understand [12]. Combined with constraints on time, resources and capacity for evidence synthesis among knowledge users, significant challenges to using systematic review evidence in healthcare decision-making exists.

In 2007, the Canadian Agency for Drugs and Technologies in Health (CADTH) in collaboration with the Cochrane Effective Practice and Organisation of Care (EPOC) Group, and the Cochrane Consumers and Communication Review Group (CC&CRG) developed *Rx for Change*, an online database of appraised and summarized systematic review evidence on various interventions to improve health professional behaviour, including prescribing and medication use (http://www.rxforchange.ca) [13]. The purpose of the database was to facilitate knowledge user access to updated systematic review evidence and, most importantly, facilitate understanding of relevant high quality systematic reviews. Target knowledge users included policymakers, decision-makers for drug plans, quality improvement organisations, non-government organisations, healthcare managers responsible for the development of programs to improve prescribing and medication use, as well as healthcare providers and consumers. Despite the introduction of initiatives to facilitate use of the database (e.g. workshops, webcasts), and positive attitudes reported towards the value of *Rx for Change* [13], use of this evidence resource has remained suboptimal, with users identifying the need for additional training. Internal information collected by CADTH liaison officers, who represent CADTH within provincial Canadian jurisdictions, indicated that users find the database overwhelming, and the educational supports, while helpful, do not allow enough time to become familiar with database structure and content. Further training opportunities to maximise how the database is used to help inform evidence-based decisions was identified as an outstanding need.

The overall objective of this research was to engage knowledge users in order to co-design, implement and evaluate a training program for facilitating use and understanding of the *Rx for Change* database. This paper describes Phase 1 of this initiative – the development of a knowledge user-informed training program for the *Rx for Change* database. Phase 2 will include implementation and an evaluation of the training program. Five medicine-focused organisations were involved; three organisations in Canada who were concerned with policy relevant to provider behaviour and two organisations in Australia who were concerned with policy relevant to consumer behaviour.

## Methods

Development of our training used an integrated knowledge translation (KT) approach [14] and was guided by the Knowledge-to-Action (KTA) framework [15]. This approach recognizes the inherent benefits to collaborating with knowledge users and including the perspectives of the target audience of an intervention in its development [14]. The KTA framework outlines the following steps as important avenues for targeting knowledge user input when developing an intervention: problem identification, contextualizing evidence and barriers assessment, and intervention development. Guided by the KTA framework, our training development process consisted of four steps: (1) key informant interviews; (2) analysis of interviews and draft training development; (3) confirmation of feasibility of the proposed training by knowledge users; and (4) final training development.

Five principal knowledge user organisations (three Canadian, two Australian) participated. Each organisation provided outreach to either health providers or consumers specific to best practices for the prescribing and use of medicines. These five organisations were recruited to participate in the study based on their interest in using *Rx for Change* on a regular basis, and each had expressed an identified need for additional *Rx for Change* training. Indeed, this project grew from the view of the organisations that the database had a lot to offer and that they were not using the database to its full potential when developing and implementing evidence-based medicine use programs within their jurisdictions. Our interest was also in ensuring the inclusion of both provider and consumer perspectives – a direct link to the two Cochrane review groups (EPOC and CC&CRG) that were involved in the development of *Rx for Change*. Having three provider-oriented organisations in Canada and two consumer-oriented organisations in Australia was a reflection of the international nature of our team (i.e. Canada and Australia) and the countries in which the two Cochrane review groups (EPOC and CC&CRG) were (at the time) situated.

Each of the organisations had a principal knowledge user who was part of the research team and was involved in the entire research process, including the development of the research protocol. The same process of intervention development was followed at all five of the organisations.

The study was approved by the research ethics boards at the Ottawa Hospital Research Institute, Ottawa, Canada, and La Trobe University, Melbourne, Australia (#20130010-01H).

### Step 1: Key informant interviews

The principal knowledge user from each organisation was asked to recommend staff members to participate in key informant interviews. Staff members’ selection for the interview was based on (1) the position of the individual within the organisation, ensuring that a diverse sample was achieved and (2) a job role appropriate to the use of *Rx for Change* ensuring that a targeted but diverse sample was achieved. A semi-structured interview guide developed by the research team and which included the knowledge users was used; the interview guide addressed the following topics: organisation characteristics, use of *Rx for Change*, use of evidence for decision-making, barriers and facilitators to using *Rx for Change*, local training needs, and preferred approaches to the training. Following these topic questions, the interview included a 15-minute session in which key informants navigated the *Rx for Change* database. They were asked to use the database to seek evidence regarding either a relevant project the informant was undertaking or a hypothetical project suggested by the interviewer. This navigation task was intended to provide additional information regarding the process of using the database that could not be collected from questioning. All interviews were audiotaped and transcribed verbatim, including the dialogue from the navigation session.

### Step 2: Analysis of data and development of draft training

A directed content analysis [16] of all transcripts was performed and NVivo Version 9 (QSR International, Cambridge, MA) was used for data management and coding. This entailed line-by-line coding into the existing interview categories (e.g. barriers to using *Rx for Change*, local training needs), followed by a thematic analysis that considered each interview category independently as well as all of the interview categories together. This method provided the opportunity to focus our attention on separate aspects of the intervention design as needed (e.g. local preferences for the training) as well as an overall thematic analysis. The line-by-line coding was conducted by one member of the research team from each country (EH in Canada, DL in Australia) and the thematic analysis was conducted by at least two members of the research team (two of HC, EH, DL). The intent of the separate country coding was to determine if there were any differing perspectives based on provider-oriented and consumer-oriented organisations. The results of the analyses were used to develop a proposed training program.

### Steps 3 and 4: Confirmation of feasibility of proposed training by knowledge users and development of final training

The project team confirmed the feasibility of the proposed training program with one target knowledge user in each organisation. These targets were the principle knowledge users that were on the research team or key leaders in the organisation. This feedback was collected verbally from each target after sharing the proposed training. Summary information from each of the targets was discussed with three members of the research team (HC, EH, DL) and final decisions were made regarding the content and delivery of the intervention. The intent of this round was to ensure that the proposed training was consistent with what was feasible for each of the organisations in terms of time and resources and perceptions of what would benefit the organisation most. This step resulted in a final training program for implementation and evaluation that was customized to the needs and audiences for each of the knowledge user organisations.

## Results

### Characteristics of key informants

Nine face-to-face key informant interviews across all three Canadian organisations (n = 1, n = 3, n = 5) and seven (one face-to-face and six telephone) interviews across two organisations in Australia (n = 3, n = 4) were completed between April and July 2013. All of the staff members who were recommended by the organisation and then approached for an interview (n = 16) agreed to participate. The average duration of the interviews was approximately 45 minutes. Key informants held their positions in their respective organisation for an average of six years (range 1–16 years) and had worked in the area of healthcare for an average of 19 years (range 1–30 years). Eight of nine Canadian and six of seven Australian key informants were aware of *Rx for Chang*e. Seven of nine Canadians, and five of seven Australians had used *Rx for Change* but none of the key informants had received any formal training in using the database. Three of nine Canadians and three of seven Australians reported having been encouraged to use the database within their organisations.

### Analysis of data and development of the training

Analysis of the transcripts revealed six key themes to guide the development of the training: (1) need for knowledge on scope and content of database; (2) developing skills for navigating the database; (3) developing skills for incorporating evidence into decision-making; (4) supporting routine use of the database; (5) need for tailoring; and (6) diversity of delivery preferences. Each is summarized below. Notably, the themes identified were consistent; there were no discernible differences found between the provider- and consumer-oriented organisations.

#### Need for knowledge

The interviews revealed a set of information about *Rx for Change* for which the key informants were either unaware of or had an incorrect knowledge of the facts. This misinformation included the type of information in *Rx for Change*, what a systematic review is, how the database is populated, how it is kept up to date, the meaning of the quality assessment, understanding of the taxonomies on which *Rx for Change* is based [17], and the value of utilising the database. The key informants were expecting *Rx for Change* to provide them with clear direction on changing prescribing/medicine use behaviours and were often frustrated by the limited practical information that they were able to find.

#### Developing skills for navigating the database

Key informants indicated in general that *Rx for Change* was not a user-friendly database, both in terms of layout and the terminology used. The general perception among key informants was that the database would be similar to other general search databases (e.g. PubMed) creating the mistaken perception that one could search the database using phrases, synonyms, limiters and/or Boolean operators to refine the relevance of the search output. When the simplistic functionality of keyword searching proved limiting, users lost interest and stopped searching.

#### Developing skills for using evidence for decision-making

Despite indicating a belief in the value of using evidence, the key informants expressed difficulty in how to find the evidence and how to incorporate the evidence found into their decision-making processes. Key informants expressed that, often, evidence was difficult to access and they were uncertain how to use the evidence into their day-to-day decision-making.

#### Routine habit

Key informants expressed the need for support to make the use of the database part of their routine activity. They believed that having reminders to use the database would be helpful.

#### Need for tailored content

Key informants expressed the need for the training to focus on issues that were currently relevant to the users’ work context.

#### Diversity of delivery preferences

Significant heterogeneity existed across organisations in terms of preferred approaches for training, with some knowledge users requesting a full day workshop and others indicating they would only access material they could engage with on their own time. Mixed feedback was received in terms of what the training should look like and ranged from a 15-minute instructional video to be viewed at the discretion of the respondents/participants, through to a 2-day workshop.

Although the thematic analysis provided valuable information on the perspectives of the knowledge users and served to guide intervention development, the thematic analysis alone was insufficient to develop the intervention. In order to facilitate the process of designing the intervention, our team engaged in a process of reviewing the thematic analysis while also considering three factors: (1) what the themes meant in terms of the content and delivery [18] of the training; (2) the need for tailoring (both in content and delivery) [19, 20]; and (3) evidence that would support the use of different strategies of the training. This approach was taken to ensure clear links between the interview themes and the training components. We felt that this process was best achieved with a 1-day face-to-face meeting between three of the researchers who were involved in the thematic analysis (HC, EH, DL) with additional input from two other team members who were not involved in the thematic analysis (DB, JG). Table 1 outlines the end result of this meeting with a description of how the themes and the information within the themes were linked to the tailored content and delivery of the intervention, and evidence.

**Table 1:** Summary of training development process – Stage 1

Theme	Descriptive details of theme	Considerations for training (content, delivery, tailoring)	Rationale (evidence to support training approach)
Need for knowledge Quote: “*I think the main barrier is knowledge. … knowledge of how it works, what it contains and for me would be having a sense of how often it is updated, how comprehensive is it, how much I can trust it, that is the kind for that key information that I am looking for. Has this done enough of the work in this area for me to use or do I have to do additional search*”	Targets expressed limited knowledge of the type of information contained within *Rx for Change*, how the database is populated, how it is kept up to date, the purpose and relevance of the quality assessment, understanding of the EPOC and CCCRG taxonomies on which *Rx for Change* is based, and the value of database	Content: Include information on as many of the deficits as possible Delivery: Fact sheets, FAQ sheets, 1:1 didactic, webinar, workshop Tailoring: Create an introduction to the workshop or webinar that can be emailed to potential participants to provide basic knowledge that some might need more than others	Knowledge: provision of information [38]
Needed skills			
1) Navigating the database Quote: “*the key one I think in terms of being challenging to find, to understand how to use the system or how to search for things*”	Issues for navigation: how to enter search terms, add limits, open or closed search, how to determine how many studies are in the review, how are individual studies assessed, how is drop down menu subdivided, what should one expect to find under each heading, why did this review show up as opposed to other reviews	Content: Use examples from participants or create standard examples. Hands-on navigation using relevant examples. Conduct comparative exercises using *Rx for Change* versus other databases to gather evidence. Use of a worksheet to facilitate navigation skills. Use screen capture of navigation of the data base that uncovers the layers and explains the components of the database Delivery: Workshop, webinar, YouTube videos Tailoring: Locally relevant examples for practice	Modelling, demonstration [38]
2) Incorporating evidence into decision-making Quote: “*if you were looking for some suggestions as to a way to structure and implementation project so go here and it will tell you what’s the most helpful, what is the quickest, or what is the whatever, but it doesn’t really do that*”.	Incorporating info into decision-making: The limitations of evidence in general related to decision-making Understanding use when evidence is there and strong and when it is not Understanding how *Rx for Change* can help them with their decision-making, how it fits with the other sources of evidence they may use	Content: Use of a guide or rubric to facilitate learning. Use of an example, group discussion Delivery: Workshop, webinar, practical and hands on Tailoring: Locally relevant examples	Modelling, demonstration [38] SUPPORT format for applying evidence [21]
Routine habit	The need to make using the database a routine aspect of every prescribing-based project	Content: The use of reminders for the database’s use, value, navigation tips. Follow-up with resources (Frequently Asked Questions, workshop presentation) and a reminder to use *Rx for Change* at 1 and 3 months post workshop Delivery: Email post-training, flyers	Reminders [42]
Need for tailoring		The tailoring required for each theme is summarized by the themes	
Diversity of delivery preferences		Content: n/a Delivery: Differing perspectives on delivery requires a multi-pronged approach in order to engage the group Tailoring: Tailor the training to the different groups by having different options for the content	

### Confirmation of feasibility of proposed training by knowledge users and development of final training

The second round of input on the proposed training was obtained from one principal knowledge user in each of the five organisations. This feedback mostly focused on available time in each organisation in terms of resources as well as input on relevant projects or upcoming program development that might be informed by evidence. The input highlighted the need to limit the total training to a 1-hour workshop as all organisations were limited in the time they could commit to staff education. Principal knowledge users also provided needed information on how best to ensure that the workshops could accommodate the plan for all participants to have access (i.e. devices and Wi-Fi) to the *Rx for Change* database during the workshop.

### Final training

Table 2 outlines details of the final training intervention using the headings content, delivery and tailoring as well as the associated themes that each aspect of the training addressed. A brief description of the contents of the intervention is included below:

1. An informational video: This 6-minute video serves as an invitation to the workshop as well as a way to provide basic background information about the database and systematic reviews. The video link is sent electronically to workshop participants who are asked to view the video prior to the workshop. A number of key informants had expressed the need for a brief and practical session, so this component of the training module aims to provide necessary background information about the database that can be viewed on participants’ own time, thus allowing for the main training session to be brief and focus on practical aspects of using and understanding the database.
2. A 60-minute face-to-face workshop: The workshop consists of a 15 minute didactic portion followed by a 30 minute skills demonstration and practice with the database, and a 15 minute discussion. The skills demonstration and practice takes the participants through the SUPPORT Tool process [21] using a current and local topic provided to the research team by the key knowledge users in each of the organisations. The SUPPORT tool process includes the following steps: Identifying the problem and/or question at hand, “What do I want to achieve?”; identifying the available options to address the problem; identifying the available resources; decision making; and implementation of most suitable options. Each step is conducted with direct connection and relevance to the local and current prescribing-based issue in the organisation. A full set of the slides is available by request through the corresponding author. The hands-on component includes (1) how to navigate the database and demonstrate the structure and different layers of *Rx for Change*, and (2) how the information within the database can be used to answer questions as related to the example or scenario. Handouts of the content and presentation are given to the participants.
3. Two post-training reminders: Reminders are sent to workshop participants at 1 and 3 months following the training and consist of an email reminding them about the workshop on *Rx for Change* and encouragement to use the database as a routine part of their work.

**Table 2:** Detailed description of delivered training with related themes

Training description	Related themes
1. Video: Invite for the workshop and foundational information on *Rx for Change* and systematic reviews Delivery: A video (prezi with audio narration) covering general information about *Rx for Change* and systematic reviews; the video was sent electronically 1 week prior to the workshop with a request for people to review it prior to attending the workshop; duration was 6 minutes Content: Quick facts about the database, information on the type of evidence included, information on when it can be used and how it can help participants in their work; an invitation to attend a free workshop and to explore *Rx for Change* on-line in a group environment was included	Need for knowledge Diversity in delivery
2. Face-to-face workshop (1 hour) Delivery: Didactic presentation (15 min) + hands on skills training (45 min) + discussion (15 min) + handouts Content of didactic presentation: *Rx for Change* was presented as part of the evidence source “puzzle” Knowledge users’ questions (as per interviews) partly guided the session’s themes (e.g. What is *Rx for Change*? Can I trust the evidence presented? What types of questions are relevant? How is *Rx for Change* different compared to other evidence sources?); the didactic part of the session focused on emphasizing “How we turn a research question into a “searchable question” in the *Rx for Change* database” Content of hands-on skills training: A pre-determined example of a research question was used to demonstrate and familiarise participants with strategic navigation strategies for the *Rx for Change* database; screenshots were used along with an audio or live presentation/demonstration; the three-question framework based on Lavis et al. [21] was used to demonstrate different ways of searching efficiently, the layout of the database, the taxonomy and the layers of the database Participants were then asked to use one of their own work examples and search the database based on the same three-question framework; this was a facilitated exercise The session was facilitated by a research team member with expertise in *Rx for Change* or an external collaborator with knowledge of *Rx for Change* Content of discussion: The incorporation and applicability of the evidence in one’s context as well as the strengths and limitations of the database and the evidence that it includes was discussed Handouts: Frequently Asked Questions and a “map” of the database layout (screenshots) were given to participants in the form of handouts after the session (for desk use)	Didactic presentation: Need for knowledge Hands on skills training: Need for skill development in navigating the database and integrating evidence into decision-making, tailoring Discussion: Knowledge and skills Handouts: Need for knowledge, variety in delivery methods
3. Post-training reminders Delivery: Emailed at 1 and 3 months post-workshop Content: Cues and/or reminders regarding the database’s use, value, updates, and navigation tips	Routine habits

## Discussion

We describe the development of a knowledge user-informed training program to increase the use of *Rx for Change*, an on-line database of quality assessed and summarized systematic reviews focused on best practice for the prescribing and use of medicines. Our process was focused on ensuring that the training incorporated the perspectives of the target knowledge users. We interviewed 16 knowledge users across two countries and five organisations in a process of information gathering and training development and clearly situated the results of the interviews in terms of the content, delivery and tailoring of the training. Next steps include implementation and evaluation.

Policy decisions involve a number of competing factors in addition to evidence [22]. In a qualitative study of how drug policymakers make decisions, Ritter et al. [23] found that evidence is but one of nine potential sources of information used to make decisions including politics, funding and public opinion. In fact, scientific evidence was found to be one of the least preferred sources due to its time consuming and contradictory nature. Policymakers by far preferred summaries of information [24, 25]. While *Rx for Change* was designed to align with these preferences through the use of evidence summaries, it might not be comprehensive enough, or indeed, even be in a preferred format to meet the needs of policymakers. It is possible that regardless of how well an on-line database of systematic reviews is designed and how well training is aligned to user needs, the lack of importance attached to scientific evidence will prevent optimal use of such resources. A better understanding of how to summarize and integrate systematic review evidence into policy decision-making is needed. Future work could include follow-up with policymakers to assess how information from sources such as *Rx for Change* impacted their policy decision-making experiences/process.

Engaging knowledge users in the training development process highlighted knowledge gaps and user information needs and preferences. Commonly referred to as integrated KT (iKT) [14], this process of engaging users in the development and testing of KT interventions has been increasing in popularity [26, 27]. As is typical to iKT efforts [28] there was a sharing of perspectives with both the research team and the knowledge users broadening their perspectives on the use of evidence and the *Rx for Change* database. However, including the perspectives of the knowledge users in training design required time and resources. The entire process of training development took approximately 1 year. Our training is more likely to be relevant to the knowledge users [15] and, therefore, potentially more effective but not without a cost. Although not designed to test this assumption, our implementation and evaluation will provide useful information on the benefits of our training.

The process of integrating a qualitative analysis of user perspectives into the design of an intervention was not straightforward. It was a challenge to make this process systematic, rigorous and replicable. The preferences for training vary widely. Although knowledge users were asked to consider what their organisation might need for database training more generally, it is not clear how different knowledge levels about the database prior to the interviews affected their stated needs for training. Improving the problem of inadequate reporting of interventions [29], as well as their design [30], is needed. Other methodology papers for KT intervention design do exist [31–34] and some outline an integrated KT approach [35], but these are not abundant. Research teams that design interventions should be encouraged to publish methods papers on their processes for intervention design. The use of tailoring and situating the training in a local context was important to our knowledge users, even across organisations that differed in roles and responsibilities. Although modest, some evidence exists for the effectiveness of tailoring KT interventions [19]. Balancing core information with tailored information is an issue that needs attention because training that is relevant has to connect with specific locales or organisational responsibilities. In addition, clarity of the benefits of tailoring would provide justification for its time consuming nature.

Different methods exist for the design of interventions aimed at changing health provider or health behaviour [32, 34, 36]. While consensus on a ‘best’ approach has not been achieved, recommendations are often made to use a theory-based approach. Our approach focused on collecting knowledge user perspectives to design the intervention and drew upon an iKT perspective. We did not, however, specifically draw on other KT or behaviour change theory to design our intervention. Although much encouragement and recommendation exists for the judicious use of theory to design interventions [30, 37, 38], theory use remains low [37]. It is likely that a range of methods are needed, both including the use of theoretical frameworks as well as specific theories for characterizing and designing behaviour change interventions such as the Capability, Opportunity and Motivation model [39]. Understanding of which method produces the best results under which conditions remains to be found. We have presented a relatively straightforward process.

Several limitations warrant mentioning. We only conducted one round of feedback. The phase to confirm feasibility of the intervention was conducted with a manager at the organisation and not with all the individuals who were interviewed. It is possible that additional rounds of feedback would have resulted in a more developed and possibly different intervention. The initial line-by-line coding into the existing interview categories (e.g. barriers information into a barriers code) was undertaken by one person, whereas the thematic analysis was undertaken by two people; therefore, we might have ended up with different coding had two people been used in the earlier stage. Finally, we focused our efforts on the iKT approach and not on other potentially informative concepts such as principles of user-centred design [40] or literature related to web-enabled resources [41]. Future efforts to understand the user experience in relation to on-line databases more broadly would be worthwhile.

## Conclusions

We have described a method of engaging knowledge users in the development of a training program to improve the use of *Rx for Change*, an on-line database of systematic reviews. Evaluation will guide future activities to optimize *Rx for Change* use by varied user groups and guide our efforts for the use of databases of evidence summaries in general.

## Abbreviations

CADTH, Canadian Agency for Drugs and Technologies in Health; CC&CRG, Cochrane Consumers and Communication Review Group; EPOC, effective practice and organisation of care; iKT, integrated knowledge translation; KT, knowledge translation; KTA, knowledge to action

## References

[1] Kleinke J (2001). The price of progress: prescription drugs in the health care market. Health Aff.

[2] Schoen C, Osborn R, Huynh PT, Doty M, Zapert K, Peugh J, Davis K. Taking the pulse of healthcare systems: experiences of patients with health problems in six countries. Health Aff. 2005;Suppl Web Exclusives:W5-509-25. 10.1377/hlthaff.w5.509 16269444

[3] Schoen C, Osborn R, How SK, Doty MM, Peugh J (2009). In chronic condition: experiences of patients with complex health care needs, in eight countries, 2008. Health Aff.

[4] Avery AJ, Sheikh A, Hurwitz B, Smeaton L, Chen Y-F, Howard R, Cantrill J, Royal S (2002). Safer medicines management in primary care. Br J Gen Pract.

[5] Arnold SR, Straus SE, Arnold S (2005). Interventions to improve antibiotic prescribing practices in ambulatory care. Cochrane Database Syst Rev.

[6] Winterstein AG, Sauer BC, Hepler CD, Poole C (2002). Preventable drug-related hospital admissions. Ann Pharmacother.

[7] Gandhi TK, Weingart SN, Borus J, Seger AC, Peterson J, Burdick E, Seger DL, Shu K, Federico F, Leape LL (2003). Adverse drug events in ambulatory care. N Engl J Med.

[8] Haynes RB, Ackloo E, Sahota N, McDonald HP, Yao X (2008). Interventions for enhancing medication adherence. Cochrane Database Syst Rev.

[9] Iuga AO, McGuire MJ (2014). Adherence and health care costs. Risk Managem Healthc Policy.

[10] Grimshaw JM, Shirran L, Thomas R, Mowatt G, Fraser C, Bero L, Grilli R, Harvey E, Oxman A, O’Brien MA (2001). Changing provider behavior: an overview of systematic reviews of interventions. Med Care.

[11] Ryan R, Santesso N, Hill S, Kaufman C, Grimshaw J (2011). Consumer‐oriented interventions for evidence‐based prescribing and medicine use: an overview of Cochrane reviews. Cochrane Database Syst Rev.

[12] Lavis JN (2006). Research, public policymaking, and knowledge-translation processes: Canadian efforts to build bridges. J Contin Educ Health Prof.

[13] Weir MC, Ryan R, Mayhew A, Worswick J, Santesso N, Lowe D, Leslie B, Stevens A, Hill S, Grimshaw JM (2010). The Rx for Change database: a first-in-class tool for optimal prescribing and medicines use. Implement Sci.

[14] Straus S, Tetroe J, Graham ID. Knowledge translation in health care: moving from evidence to practice. Chichester: John Wiley & Sons; 2013.

[15] Graham ID, Logan J, Harrison MB, Straus SE, Tetroe J, Caswell W, Robinson N (2006). Lost in knowledge translation: time for a map?. J Contin Educ Heal Prof.

[16] Hsieh HF, Shannon SE (2005). Three approaches to qualitative content analysis. Qual Health Res.

[17] Lowe D, Ryan R, Santesso N, Hill S (2011). Development of a taxonomy of interventions to organise the evidence on consumers’ medicines use. Patient Educ Couns.

[18] Webb T, Joseph J, Yardley L, Michie S (2010). Using the internet to promote health behavior change: a systematic review and meta-analysis of the impact of theoretical basis, use of behavior change techniques, and mode of delivery on efficacy. J Med Internet Res.

[19] Shaw B, Cheater F, Baker R, Gillies C, Hearnshaw H, Flottorp S, Robertson N (2005). Tailored interventions to overcome identified barriers to change: effects on professional practice and health care outcomes. Cochrane Database Syst Rev.

[20] Wensing M, Oxman A, Baker R, Godycki-Cwirko M, Flottorp S, Szecsenyi J, Grimshaw J, Eccles M (2011). Tailored Implementation For Chronic Diseases (TICD): a project protocol. Implement Science.

[21] Lavis JN, Boyko JA, Oxman AD, Lewin S, Fretheim A (2009). SUPPORT Tools for evidence-informed health Policymaking (STP) 14: Organising and using policy dialogues to support evidence-informed policymaking. Health Res Policy Sys.

[22] Lavis JN, Posada FB, Haines A, Osei E (2004). Use of research to inform public policymaking. Lancet.

[23] Ritter A (2009). How do drug policy makers access research evidence?. Int J Drug Policy.

[24] Dobbins M, DeCorby K, Robeson P, Husson H, Tirilis D, Greco L (2010). A knowledge management tool for public health: health-evidence.ca. BMC Public Health.

[25] Armstrong R, Waters E, Dobbins M, Anderson L, Moore L, Petticrew M, Clark R, Pettman TL, Burns C, Moodie M (2013). Knowledge translation strategies to improve the use of evidence in public health decision making in local government: intervention design and implementation plan. Implement Sci.

[26] Ellen ME, Léon G, Bouchard G, Ouimet M, Grimshaw JM, Lavis JN (2014). Barriers, facilitators and views about next steps to implementing supports for evidence-informed decision-making in health systems: a qualitative study. Implement Sci.

[27] Ellen ME, Lavis JN, Ouimet M, Grimshaw J, Bédard P-O (2011). Determining research knowledge infrastructure for healthcare systems: a qualitative study. Implement Sci.

[28] Graham ID, Tetroe J (2007). Some theoretical underpinnings of knowledge translation. Acad Emerg Med.

[29] Hoffmann TC, Glasziou PP, Boutron I, Milne R, Perera R, Moher D, Altman DG, Barbour V, Macdonald H, Johnston M (2014). Better reporting of interventions: template for intervention description and replication (TIDieR) checklist and guide. BMJ.

[30] Campbell NC, Murray E, Darbyshire J, Emery J, Farmer A, Griffiths F, Guthrie B, Lester H, Wilson P, Kinmonth AL (2007). Designing and evaluating complex interventions to improve health care. BMJ.

[31] Kolehmainen N, Francis JJ (2012). Specifying content and mechanisms of change in interventions to change professionals’ practice: an illustration from the Good Goals study in occupational therapy. Implement Sci.

[32] Curran GM, Mukherjee S, Allee E, Owen RR (2008). A process for developing an implementation intervention: QUERI Series. Implement Sci.

[33] Van Bokhoven M, Kok G, Van der Weijden T (2003). Designing a quality improvement intervention: a systematic approach. Qual Saf Health Care.

[34] French SD, Green SE, O’Connor DA, McKenzie JE, Francis JJ, Michie S, Buchbinder R, Schattner P, Spike N, Grimshaw JM (2012). Developing theory-informed behaviour change interventions to implement evidence into practice: a systematic approach using the Theoretical Domains Framework. Implement Sci.

[35] Cabassa LJ, Druss B, Wang Y, Lewis-Fernández R (2011). Collaborative planning approach to inform the implementation of a healthcare manager intervention for Hispanics with serious mental illness: a study protocol. Implement Sci.

[36] Bartholomew LK, Parcel GS, Kok G, Gottlieb NH. Planning health promotion programs: an intervention mapping approach. San Fransisco, CA: John Wiley & Sons; 2011.

[37] Colquhoun HL, Brehaut JC, Sales A, Ivers N, Grimshaw J, Michie S, Carroll K, Chalifoux M, Eva KW (2013). A systematic review of the use of theory in randomized controlled trials of audit and feedback. Implement Sci.

[38] Michie S, Johnston M (2012). Theories and techniques of behaviour change: developing a cumulative science of behaviour change. Health Psychol Rev.

[39] Michie S, van Stralen MM, West R (2011). The behaviour change wheel: a new method for characterising and designing behaviour change interventions. Implement Sci.

[40] Maguire M. Methods to support human-centred design. Int J Hum-Comput St. 2001;55(4):587–634.

[41] Mathew D, McKibbon KA, Lokker C, Colquhoun H (2014). Engaging with a Wiki related to knowledge translation: a survey of WhatisKT Wiki users. J Med Internet Res.

[42] Shojania KG, Jennings A, Mayhew A, Ramsay C, Eccles M, Grimshaw J. Effect of point-of-care computer reminders on physician behaviour: a systematic review. CMAJ. 2010;182(5):E216–25. 10.1503/cmaj.090578 PMC2842864 20212028

